# Novel insights related to the rise of KPC-producing *Enterobacter cloacae* complex strains within the nosocomial niche

**DOI:** 10.3389/fcimb.2022.951049

**Published:** 2022-10-24

**Authors:** Camila A. Knecht, Natalia García Allende, Verónica E. Álvarez, Barbara Prack McCormick, Mariana G. Massó, María Piekar, Josefina Campos, Bárbara Fox, Gabriela Camicia, Anahí S. Gambino, Ana Carolina del Valle Leguina, Nicolás Donis, Liliana Fernández-Canigia, María Paula Quiroga, Daniela Centrón

**Affiliations:** ^1^ Laboratorio de Investigaciones en Mecanismos de Resistencia a Antibióticos, Instituto de Investigaciones en Microbiología y Parasitología Médica, Facultad de Medicina, Universidad de Buenos Aires - Consejo Nacional de Investigaciones Científicas y Técnicas (IMPaM, UBA-CONICET), Ciudad Autónoma de Buenos Aires, Argentina; ^2^ Servicio de Infectología y Epidemiología Hospitalaria, Hospital Alemán, Ciudad Autónoma de Buenos Aires, Buenos Aires, Argentina; ^3^ Facultad de Ciencias Agrarias, Universidad Nacional de Lomas de Zamora (FCA, UNLZ), Lomas de Zamora, Argentina; ^4^ Plataforma de Genómica y Bioinformática, Instituto Nacional de Enfermedades Infecciosas-Administración Nacional de Laboratorios e Institutos de Salud (INEI-ANLIS), Ciudad Autónoma de Buenos Aires, Argentina; ^5^ Departamento de Microbiología, Hospital Alemán, Ciudad Autónoma de Buenos Aires, Buenos Aires, Argentina

**Keywords:** *Klebsiella pneumoniae*, *Enterobacter cloacae* complex, carbapenem-resistance, *bla*_KPC-2_, Argentina

## Abstract

According to the World Health Organization, carbapenem-resistant *Enterobacteriaceae* (CRE) belong to the highest priority group for the development of new antibiotics. Argentina-WHONET data showed that Gram-negative resistance frequencies to imipenem have been increasing since 2010 mostly in two CRE bacteria: *Klebsiella pneumoniae* and *Enterobacter cloacae* Complex (ECC). This scenario is mirrored in our hospital. It is known that *K. pneumoniae* and the ECC coexist in the human body, but little is known about the outcome of these species producing KPC, and colonizing or infecting a patient. We aimed to contribute to the understanding of the rise of the ECC in Argentina, taking as a biological model both a patient colonized with two KPC-producing strains (one *Enterobacter hormaechei* and one *K. pneumoniae*) and *in vitro* competition assays with prevalent KPC-producing ECC (KPC-ECC) versus KPC-producing *K. pneumoniae* (KPC-Kp) high-risk clones from our institution. A KPC-producing *E. hormaechei* and later a KPC-Kp strain that colonized a patient shared an identical novel conjugative IncM1 plasmid harboring *bla*_KPC-2_. In addition, a total of 19 KPC-ECC and 58 KPC-Kp strains isolated from nosocomial infections revealed that high-risk clones KPC-ECC ST66 and ST78 as well as KPC-Kp ST11 and ST258 were prevalent and selected for competition assays. The competition assays with KCP-ECC ST45, ST66, and ST78 versus KPC-Kp ST11, ST18, and ST258 strains analyzed here showed no statistically significant difference. These assays evidenced that high-risk clones of KPC-ECC and KPC-Kp can coexist in the same hospital environment including the same patient, which explains from an ecological point of view that both species can exchange and share plasmids. These findings offer hints to explain the worldwide rise of KPC-ECC strains based on the ability of some pandemic clones to compete and occupy a certain niche. Taken together, the presence of the same new plasmid and the fitness results that showed that both strains can coexist within the same patient suggest that horizontal genetic transfer of *bla*_KPC-2_ within the patient cannot be ruled out. These findings highlight the constant interaction that these two species can keep in the hospital environment, which, in turn, can be related to the spread of KPC.

## Introduction

Since 2017, the World Health Organization has classified pathogens depending on their priority for the development of new antibiotics as critical, high, and medium (World Health Organization, 2017). Carbapenem-resistant *Enterobacteriaceae* (CRE) were categorized as critical priority pathogens. This group includes bacteria that have become resistant to the best antibiotic options treatment available: carbapenems and third-generation cephalosporins. These bacteria pose a threat in healthcare facilities, especially among patients whose care requires invasive devices (Wang et al., 2016). In Argentina, carbapenem-resistant isolates rose from 10% to 32.7% in the case of *K. pneumoniae* and from 5% to 12% in the case of *Enterobacter cloacae* Complex (ECC) from 2010 until 2021 (Red WHONET Argentina). Accordingly, ECC has been reported as the second most common CRE in several countries (Tavares et al., 2015; Jia et al., 2018; Annavajhala et al., 2019; Falco et al., 2021; Hansen, 2021), with *E. cloacae* and *E. hormaechei* being the prevalent multidrug-resistant (MDR) clinical isolates (Annavajhala et al., 2019). *Enterobacter hormaechei* is part of the ECC together with 22 other species that are closely genotypically related, and little is known about its fitness within the nosocomial environment (Davin-Regli et al., 2019). Total genome sequences of various *Enterobacter* spp. have shown that *E. hormaechei* has often been misidentified by routine identification techniques (Davin-Regli et al., 2019). Therefore, its importance in the clinical environment could have been underestimated; however, outbreaks of *E. hormaechei* have been reported in the past (Campos et al., 2007; Paauw et al., 2009).

Among the plasmid-born resistance mechanisms that account for carbapenem resistance, the production of KPC has remained predominant (Bonomo et al., 2018). The most common variants of the gene that codifies for KPC are *bla*_KPC-2_ and *bla*_KPC-3_ (Brandt et al., 2019), which have become endemic in several countries (Frost et al., 2019). Argentina is among these countries, and apart from clinical isolates, *bla*_KPC-2_ has also been recently detected in sewage (Ghiglione et al., 2021). The *bla*_KPC_ genes have been found in more than 257 different representative KPC plasmids (Brandt et al., 2019) belonging to diverse Inc groups and sizes, and with several features that account for their success. Carbapenem-resistant (CR) *Klebsiella pneumoniae* (CRKP) strains carrying KPC (KPC-Kp) have been long known to represent a threat to human health causing severe infections that are difficult to treat (Heiden et al., 2020). Also, the ECC has lately awoken interest due to its increasing resistance to carbapenems codified by several genes found in isolates all over the globe (Annavajhala et al., 2019). Outbreaks take place mainly in low- and middle-income countries (BARNARDS Group et al., 2021) and are particularly dangerous for children and newborns (Girlich et al., 2021). Unlike KPC-Kp, not only stable *bla*_KPC_–high-risk clones associations account for the spread of KPC-ECC but also the acquisition of plasmids by diverse clones (Annavajhala et al., 2019). To be considered high-risk clones, the lineages must meet several characteristics: to be globally distributed, to possess several acquired antimicrobial resistance genes, to be able to colonize and persist in hosts for long periods, to be transmitted effectively among hosts, to cause severe and/or recurrent infections, and to have enhanced pathogenicity and fitness (Mathers et al., 2015).

Fitness is a fundamental notion in evolutionary biology. When compared with their less-fit competitors, genotypes with better fitness tend to produce more offspring and hence increase in frequency over time (Wiser and Lenski, 2015). High-risk clones are likely to have advantageous biological traits that boost their fitness, giving them an evolutionary advantage over other isolates of the same species (Pitout and Finn, 2020). Such traits provide them with the capacity to outperform competing bacteria and to establish as the dominant component of the bacterial community. Even though there are several methods to quantify microbial fitness, the approach that most closely corresponds to the meaning of fitness in evolutionary theory uses a competition assay (Wiser and Lenski, 2015). Competition assays between isogenic strains are common (Sander et al., 2002; Guo et al., 2012), but much less is explored about lineages or interspecies competitions that share the same ecological niche (Hafza et al., 2018; Ávarez et al., 2020).

The aim of this study was to investigate the interplay of KPC-ECC challenged with prevalent high-risk KPC-Kp clones from our institution including strains isolated from the same patient to understand if fitness contributes to the success of KPC-ECC within the nosocomial niche.

## Results

### Epidemiology of carbapenem-resistant *Enterobacteriaceae* strains isolated from nosocomial infections from October 2018 until December 2020 in our institution

From October 2018 until December 2020, a study with surveillance purposes identified 153 CRE strains isolated from nosocomial infections in our institution. This survey revealed that *K. pneumoniae* accounted for 85% of CRE, 12% were the ECC, and the remaining 3% were other CRE species. Whole-genome sequencing (WGS) of KPC-Kp (*n* = 58) and KPC-ECC (*n* = 19) nosocomial strains was sequenced by Illumina MiSeq-I (*n* = 77). Their MLST profiles were assigned using the pubMLST database (**Table S1**; Jolley et al., 2018). We identified that high-risk clones KPC-Kp ST258 and ST11, and KPC-ECC ST66 and ST78 were found among infected patients (**Table S1**). Also, for the taxonomical identification of ECC strains, we combined the results obtained with rMLST, which identified the HA2pEho, HAC11Eho, and HA58Eho strains as *E. hormaechei*, with those obtained by Kraken 2 (Wood et al., 2019); the same outcome was obtained with some extra information about the subspecies and the absence of contaminations (**Table S7**). By ANI (Jain et al., 2018) and *in silico* DNA–DNA hybridization (Meier-Kolthoff et al, 2022), we obtained additional information on the subspecies, identifying *E. hormaechei* HA2pEho ST45, HAC11Eho ST78, and HA58Eho ST66 as belonging to different subspecies (**Tables S8 and S9**). These results were also in agreement with those obtained with Kraken 2. The KPC-producing *E. hormaechei* HAC11Eho subspecies *hoffmannii* (ST78), *E. hormaechei* HA58Eho subspecies *xiangfangensis* (ST66), *K. pneumoniae* HA3pKpn (ST258), and *K. pneumoniae* HA15pKpn (ST11) strains from this survey were chosen for fitness assays.

### Novel and conjugative IncM1 pDCCK1-KPC plasmid carrying *bla*_KPC-2_ shared by both KPC-producing strains colonizing the same patient

On 20 November 2018, a 71-year-old man (patient M71) was admitted to our hospital with febrile syndrome (**Figure S1**). After 1 week of hospitalization, surgical debridement of sacral pressure ulcer was performed. A carbapenemase-producing strain was isolated from a vital tissue wound swab. After a new hospitalization in January 2019, a rectal swab was taken for surveillance purposes; a carbapenem-resistant strain was isolated. Through WGS, the first colonizing strain was identified as *E. hormaechei* subspecies *steigerwaltii* belonging to the ST45 (HA2pEho). The second strain was identified as KPC-Kp belonging to the sequence type ST18 (HA7pKpn).

**Figure 1:**
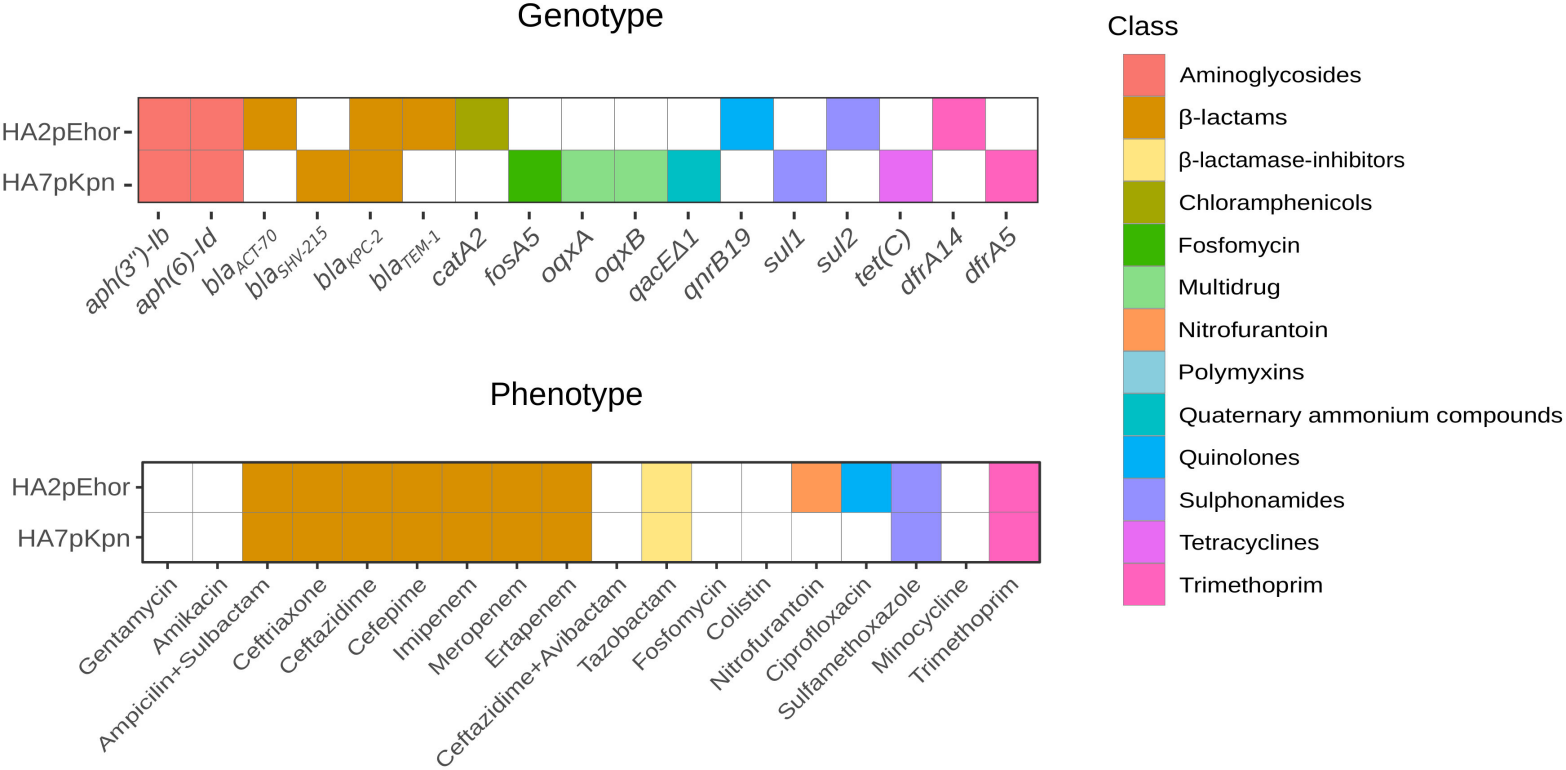
Antibiotic resistance genes and antibiotype profiles of *E. hormaechei* HA2pEho, *K. pneumoniae*, and HA7pKpn strains isolated from colonizations. Colored cell means presence and different colors indicate the antibiotic class for which the ARG codifies. The lower panel shows the resistance phenotype, and the upper panel shows the resistance genotype. The figure was made in R using the package ggplot2.

The outcome of the search for antibiotic resistance genes (ARGs) and the determination of antibiotype profiles are shown in **Figure 1**. Genome analysis and phenotypic resistance profile showed that both strains were MDR, i.e., resistant to more than three classes of antibiotics but still susceptible to more than two classes of antibiotics (Magiorakos et al., 2012). Apart from *bla*_KPC-2_, two other genes were shared by both strains, *aph(3’’)-Ib* and *aph(6)-Id*, conferring resistance to streptomycin. Besides the ARG they had in common, *E. hormaechei* HA2pEho carried *bla*_ACT-70_ (naturally harbored by the species), *bla*_TEM-1_, *catA2, qnrB19*, and *sul2*. The gene cassette *dfrA14* was identified in the variable region of a class 1 integron. *K. pneumoniae* HA7pKpn carried *bla*_SHV-215_ (naturally harbored by the species), *fosA5*, *oqxA*, *oqxB*, and *tet*(C). In addition, the gene cassette *dfrA5* was found within the variable region of a clinical class 1 integron with the 3´-conserved sequence harboring *qacEΔ1* and *sul1*. Minimum inhibitory concentrations (MICs) are shown in **Table S2**.

**Figure 2:**
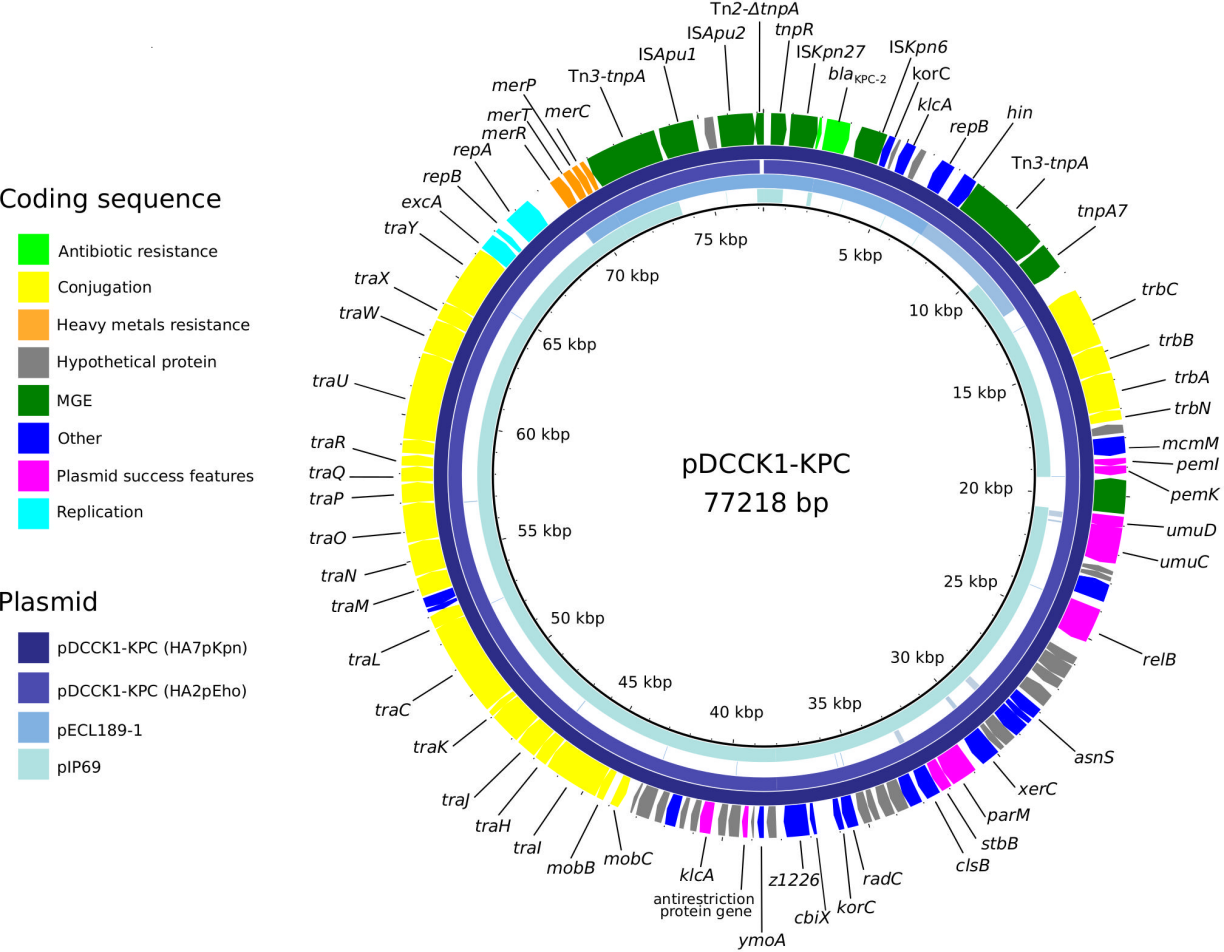
Fitness assays between KPC-ECC and KPC-Kp clones. KPC-*E. hormaechei* ST45 (HA2pEho), ST66 (HA58Eho), and ST78 (HAC11Eho) were set to compete with KPC-*K. pneumoniae* ST11 (HA15pKpn), ST18 (HA7pKpn), and ST258 (HA3pKpn). *E. hormaechei* ST45 and *K. pneumoniae* ST18 are the sequenced strains described in this study, and the other STs are high-risk clones found in our institution during a surveillance program (**Table S1**). In the cases where the values are above zero, it can be interpreted that ECC clones outcompete *K. pneumoniae* clones. The figure was made in R using the package ggplot2.

Genome analysis revealed that *E. hormaechei* HA2pEho and *K. pneumoniae* HA7pKpn strains carried several plasmids (**Table S3**). A virulence multireplicon IncHI1B/IncFIB plasmid was found in *K. pneumoniae* HA7pKpn, and a Col(pHAD28), an IncFIB(pECLA), an IncFII(pECLA), and a pKP1433 were found in *E. hormaechei* HA2pEho. In addition, a novel IncM1 plasmid named pDCCK1-KPC carrying *bla*_KPC-2_ was identical in both strains (**Figure 2**). In both cases, the whole sequence of the plasmid pDCCK1-KPC was on a single contig. In the case of *E. hormaechei* HA2pEho, the contig was 76,978 bp long, and in the case of *K. pneumoniae* HA7pKpn, it was 77,218 bp. The contiguousness of the extremes of the contigs was verified by PCR using specially designed primers. The best hit of pDCCK1-KPC against the BLAST database was plasmid pIP69 (MN626603.1) with 81% of cover and 99.98% identity. This plasmid was isolated in 1969 from a *Salmonella paratyphi* strain (Chabbert et al., 1972), and in comparison with pDCCK1-KPC, pIP69 lacked the *bla*_KPC-2_ gene and its flanking sequences (16,008 bp) (Neil et al., 2020). Although the plasmid pECL189-1 (CP047966.1) covers the whole region, which was absent in plasmid pIP69, the genetic arrangement was different, and the genes, in that case, were not contiguous. Apart from the *bla*_KPC-2_ gene, the pDCCK1-KPC plasmid carried several genes that likely account for its success such as a mercury resistance island, the gene *parM*, a toxin–antitoxin system *pemI–pemK*, and the *umuCD* operon. Conjugation assays were carried out between *E. hormaechei* HA2pEho and *K. pneumoniae* HA7pKpn as donor strains and *Escherichia coli* J53 as a recipient strain. Both assays were positive, confirming that pDCCK1-KPC can be horizontally transferred. Determination of MIC of transconjugants revealed that pDCCK1-KPC only transfers resistance to β-lactams including carbapenems (**Table S2**). This result is in agreement with the composition of pDCCK1-KPC that only carries one ARG, the *bla*_KPC-2_ gene.

A genetic platform of 17,092 bp involved in the dissemination of *bla*_KPC-2_ was identified as Tn*3*-IS*Apu1*-IS*Apu2*-IS*Kpn27*-*bla*_KPC-2_-IS*Kpn6*-*korC*-orf*-klcA-repB-hin-*Tn*3* in pDCCK1-KPC showing some differences with sequences available at the NCBI database (**Figure S2**). A very closely related core platform Tn*3*-IS*Kpn27*-Δ*bla*_TEM-1_-*bla*_KPC-2_-IS*Kpn6*-*korC*-orf*-klcA-repB* was described by Shen et al. (2009) and was later called variant 1. Also, a similar genetic platform was described in Argentina in 2011 (Gomez et al., 2011), in 2018 (De Belder et al., 2018), and more recently in 2021 (Ghiglione et al., 2021), whereas an identical platform was found in our institution (Knecht et al, 2022). In the genetic platform of both *K. pneumoniae* HA7pKpn and *E. hormaechei* HA2pEho, Δ*bla*_TEM-1_ was missing, and nucleotides between IS*Kpn27* and *bla*_KPC-2_ were only 254 bp. Moreover, a related genetic platform with the gene *hin* upstream from *repB*, more similar to our platform, was found by Dong et al. (2020). In all these cases, the genetic platform carrying *bla*_KPC-2_ was in IncP or IncR plasmids, as the main difference with pDCCK1-KPC, which corresponded to an IncM1 replicon. Although these genetic platforms are not the most frequent for the dissemination of *bla*_KPC-2_, they were identified in clinical and environmental isolates on a global scale (**Table S4**). The sequences found in the NCBI database that were most similar to the genetic platform found in pDCCK1-KPC covered 13,475 bp of the platform (**Table S4**). This search resulted in 111 hits, 89 of which were *Enterobacteriaceae*, and among them, 73 were *K. pneumoniae* and 2 were *E. cloacae*. Other bacteria belonging to different taxa with 99%–100% of query cover were two *Pseudomonas aeruginosa* and eight *Aeromonas* spp.

### *In vitro* competition between high-risk clones of KPC-producing strains of *Enterobacter cloacae* Complex and *Klebsiella pneumoniae*

To understand the interplay in the success of the two prevalent CRE in the nosocomial niche from our institution, we compared the fitness of prevalent high-risk clones of KPC-ECC and KPC-Kp strains. The study of clonal competition was carried out in a biological model without antibiotic pressure in order to replicate what can happen in the hospital environment, in which there are niches where an antibiotic pressure is not exerted directly, for example, on abiotic surfaces.

**Figure 3:**
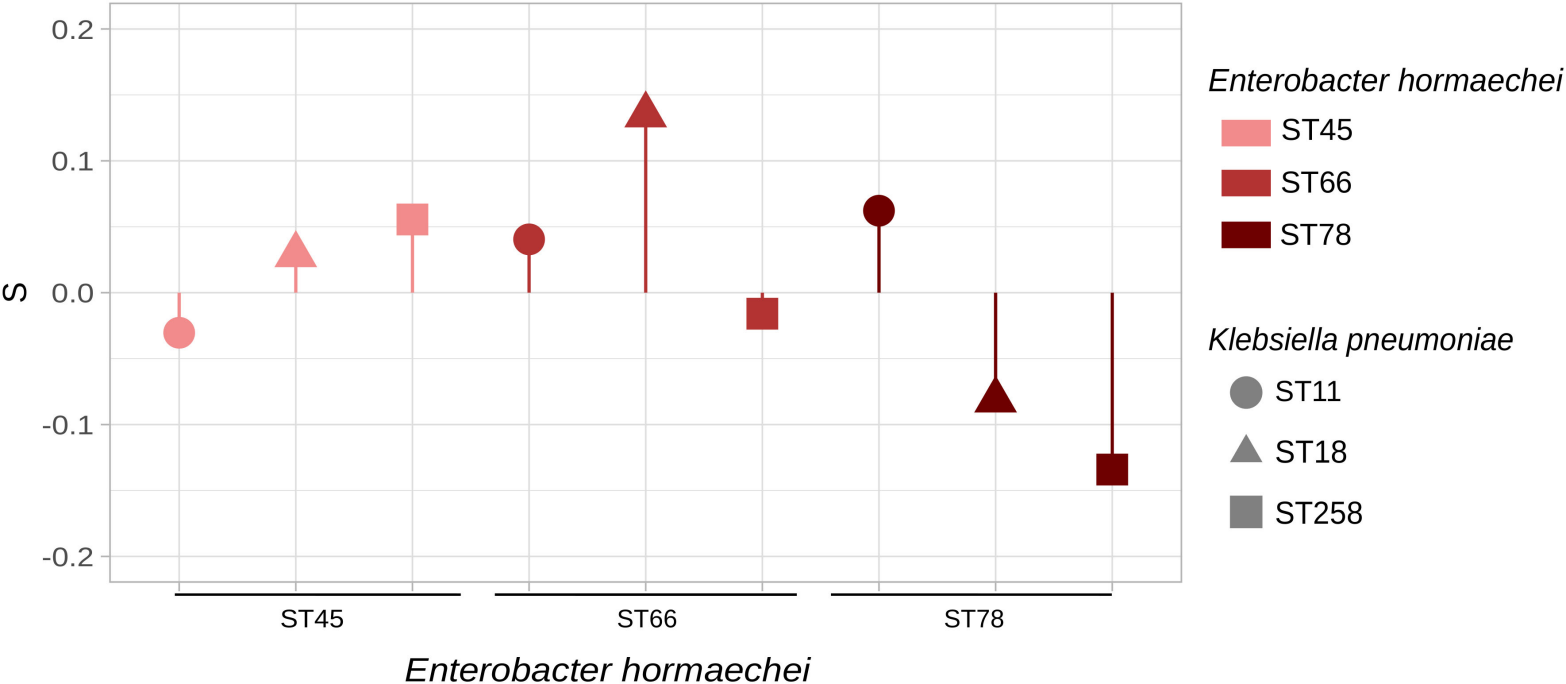
Plasmid pDCCK1-KPC from *E. hormaechei* HA2pEho and *K. pneumoniae* HA7pKpn strains and genetic platform of *bla*_KPC-2_ *.*Alignment of the novel plasmid pDCCK1-KPC against pIP69 (MN626603.1) and pECL189-1 (CP047966.1). The figure was made using BRIG.

We carried out *in vitro* competition of KPC-producing *E. hormaechei* HA2pEho ST45, *E. hormaechei* HA58Eho ST66, and *E. hormaechei* HAC11Eho ST78 versus *K. pneumoniae* HA15pKpn ST11, *K. pneumoniae* HA7pKpn ST18, and *K. pneumoniae* HA3pKpn ST258 in the absence of antimicrobial pressure (**Figure 3**; **Tables S5** and **S6**). In addition, maintenance assays of *bla*_KPC-2_ showed that this gene remains present in the absence of selective pressure for all these strains during the time of competitions. Both *E. hormaechei* ST66 and ST78, and *K. pneumoniae* ST258 and ST11 represent high-risk international clones and are also the most frequently found in our institution, in contrast to the other high-risk clone analyzed here, *E. hormaechei* ST45 (**Table S1**). Taking into account the clonal competitions involving *E. hormaechei* HA2pEho ST45, with *K. pneumoniae* HA7pKpn ST18 or with *K. pneumoniae* HA3pKpn ST258, we found that *E. hormaechei* HA2pEho ST45 showed a positive S value and fitness cost of −2.874 and −5,981%, respectively. These results indicate that *E. hormaechei* HA2pEho ST45 has a competitive advantage over these two *K. pneumoniae* ST18 and ST258 isolates. The opposite was shown for the clonal competition of *E. hormaechei* HA2pEho ST45 with *K. pneumoniae* HA15pKpn ST11. For the clonal competitions of *E. hormaechei* HAC11Eho ST78 with the three *K. pneumoniae* ST11, ST18, and ST258 strains, the S values and fitness costs obtained were opposite of those obtained for *E. hormaechei* HA2pEho ST45. The clonal competitions carried out showed only tendencies, since none of the results obtained were statistically significant. The clearer differences were obtained for *E. hormaechei* ST66 over *K. pneumoniae* ST18 and for *K. pneumoniae* ST258 over *E. hormaechei* ST78, which showed a fitness cost >10% (13,919% and −13,476, respectively). Previous studies reported that the greater the difference in growth rate between two strains, the greater the bacterial load difference over time (Guo et al., 2012). This fact was not confirmed in the case of competitions with *E. hormaechei* ST45, ST66, and ST78, and the *K. pneumoniae* ST11, ST18, and ST258 strains analyzed here. This indicates that competitions between clones have emerging properties resulting from their interactions while growing in the same niche.

Although general comparisons can shed light on the reasons for the prevalence of particular clones, the acquired antimicrobial resistance and virulence genes of a particular strain will also determine why it surpasses another under specific conditions. It has been proposed that differences in antimicrobial resistance and virulence factors such as secretion systems should be under consideration altogether and that even small genomic modifications play a role in determining the clonal competence of pathogens (Álvarez et al., 2020). Therefore, we investigated the presence of secretion systems in the two KPC strains isolated from patient M71. Twelve core genes (*gspCDEFGHIJKLMNO*) of a type II secretion system were identified in both strains colonizing patient M71. *K. pneumoniae* HA7pKpn carried type I and type II secretion system components, and *E. hormaechei* HA2pEho also encoded for proteins related to secretion system type IV. The type I secretion system was represented by the genes *hlyB*, *hlyD*, *prsE*, *tolC*, *macA*, and *macB* in HA2pEho and by *tolC, macA*, and *macB* in *K. pneumoniae* HA7pkpn. The type IV secretion system is related to the conjugation system of bacteria and directly transfers effector proteins to the host cytosol through a central pore (Voth et al., 2012). All the genes of the *virB*/*D4* complex that constitute one type of secretion system IVA were found in *E. hormaechei* HA2pEho except for *virB7* and *virD4.*

## Discussion

Our results contribute to the understanding of both the dissemination of the most clinically relevant carbapenemase, *bla*_KPC-2_ in the ECC, and the role of fitness of high-risk clones in the rise of KPC-ECC within the nosocomial environment in colonized as well as in infected patients. Since phenotypic methods or even the traditionally employed 16S rRNA gene typing is insufficient to resolve the species identification of the ECC strains (Annavajhala et al., 2019), little is known concerning their dissemination in Latin America. The molecular survey performed during the period from October 2018 until December 2020 at our institution allowed us to infer the first data on circulating clones in our geographical region, which detected high-risk clones *E. hormaechei* ST45 (subspecies *steigerwaltii*), ST66 (subspecies *xiangfangensis*), and ST78 (subspecies *hoffmannii*). Interestingly, the same STs were also the most prevalent found among carbapenem-resistant ECC (CRECC) isolates in France, but in that case, the carbapenemase genes responsible for the phenotype were alleles of the gene *bla*_VIM_ (Emeraud et al., 2022). As expected, despite constant changes regarding nomenclature within the ECC, *E. cloacae* and *E. hormaechei* continue to prevail among multidrug clinical isolates (Annavajhala et al., 2019). *E. hormaechei* ST171, ST114, and ST66 belong to CC114 and have been found to be widespread among global CRECC isolates from several countries (Peirano et al., 2018). On the other hand, *E. hormaechei* ST78 was identified as a high-risk clone among both extended-spectrum β-lactamase-producing ECC and CRECC (Gomez-Simmonds et al., 2018; Emeraud et al., 2022). *E. hormaechei* ST45 is recognized as a high-risk clone (Izdebski et al., 2015) reported in Australia (Sidjabat et al., 2015), Colombia (Falco et al., 2021), Chile (Wozniak et al., 2021), Germany (Heiden et al., 2020), Spain (Fernández et al., 2015), and other countries in Europe (Izdebski et al., 2015). As a whole, these results suggest that the establishment of successful high-risk clones of *E. hormaechei* in the nosocomial niche provides the opportunity to evolve to MDR phenotypes mediated by the acquisition and maintenance of diverse plasmids, which may have been substantial contributors to the continuous rise of CRECC.

The gene *bla*_KPC-2_ was in the same conjugative plasmid in both strains from inpatient M71. The novel plasmid that we called pDCCK1-KPC belongs to the IncM1 incompatibility group. According to a review on *bla*_KPC_ plasmids (Brandt et al., 2019), IncM plasmids were unusual at least until 2019, when just 7 out of 435 plasmids belonged to this incompatibility group. Plasmid pDCCK1-KPC is a novel rearrangement between plasmids leading to the acquisition of a new KPC-2 genetic platform by an IncM1 plasmid. These multiple events of loss and/or gain of mobile genetic elements that also include insertions, deletions, or homologous recombination coincide with what was identified for the dissemination of *bla*_KPC-2_ in previous works. Similar plasmids show traces of multiple events, with partial mobile genetic elements scattered throughout its genetic platform (Botts et al., 2017; Brandt et al., 2019; Ghiglione et al., 2021).

It cannot be ruled out that pDCCK1-KPC might have been transferred from *E. hormaechei* HA2pEho to *K. pneumoniae* HA7pKpn within the patient, although it is not possible to confirm it. Putative HGT of *bla*_KPC-2_ has been previously reported to have taken place intrapatient (Anchordoqui et al., 2015; Wozniak et al., 2021). The study by Anchordoqui et al. (2015) was also carried out in Argentina, and HGT was reported to have taken place between *E. coli* and *K. pneumoniae*, *E. cloacae* and *K. pneumoniae*, and *Citrobacter freundii* and *Klebsiella oxytoca.* In this case, the STs of strains were not determined, and plasmids belonged to different incompatibility groups including IncM/L but were not sequenced.

A very closely related genetic platform carrying *bla*_KPC-2_ was found contemporary to strains isolated from inpatient M71 in environmental strains *Enterobacter absuriae* WW14A and *Klebsiella quasipneumoniae* subsp. *quasipneumoniae* WW19C, which were recovered from the same sewage network of our institution in 2018 (Ghiglione et al., 2021). Therefore, our study also reinforces the need for studying the spread of critical acquired ARG within the One Health perspective. Since it is not possible to identify the direction of the ARG flow, the role of the environment either as a source or as a reservoir of the *bla*_KPC-2_ gene is confirmed in our findings. The platform Tn*3-*IS*Apu1-*IS*Apu2-*IS*Kpn27-bla*_KPC-2_ *-*IS*Kpn6-korC-*orf*-klcA-repB-hin-*Tn*3* (17092 bp) was identical to the one recently found in another clinical isolate from our institution (Knecht et al., 2022). In comparison with the platform in sewage strains, both its location in a different plasmid and the lack of Δ*bla*_TEM-1_ highlight the ability of *bla*_KPC-2_-flanking sequences to evolve, extending the spreading range.

The competition assays with *E. hormaechei* ST45, ST66, and ST78, and the *K. pneumoniae* ST11, ST18, and ST258 strains analyzed here showed only slight differences in the values obtained. The results obtained for the clonal competitions of the three high-risk clones of *E. hormaechei* with no statistically significant difference with the *K. pneumoniae* ST11, ST18, and ST258 strains analyzed could explain in part the co-occurrence of KPC-Kp and KPC-ECC in clinical isolates. As a consequence, these species can share the same plasmids such as pDCCK1-KPC as the main contributors to the global spread of KPC.

The fact that high-risk international clones of KPC-ECC and KPC-Kp can coexist successfully within the nosocomial niche is in agreement with the steady rise of KPC-ECC observed in Argentina since 2010 (WHONET Argentina Network) and other countries like Brazil (Tavares et al., 2015), Colombia (Falco et al., 2021), USA (Annavajhala et al., 2019; Hansen, 2021), China (Jia et al., 2018), Portugal, and India (Center for Disease Dynamics, Economics & Policy). Accordingly, a decrease in KPC-ECC strains in our hospital is not expected despite the prevalence and fruitful spread of high-risk international clones of KPC-Kp ST258 and ST11. Since both these high-risk clones are disseminated worldwide, a replica of this scenario is likely to occur in other regions.

Considering that fitness studies have previously helped to understand the emergence of relevant antimicrobial-resistant lineages (Luo et al., 2005; Otto, 2013; Ávarez et al., 2020; Hertz et al., 2022), it would be interesting to deeply investigate the interplay of most common KPC-producing clones from other institutions. Studying the ecological behavior of high-risk clones coexisting within the same hospital and their changing epidemiological patterns over time could contribute to identifying possible competitive emerging clones as well as prevent the further spread of KPC-producing strains.

## Materials and methods

### Bacterial strains and antibiotic susceptibility assays

Strains *E. hormaechei* HA2pEho and *K. pneumoniae* HA7pKpn were colonizing strains isolated from the same patient as indicated in the *Results* section. The rest of the strains were part of a surveillance program of CRE infecting strains carried out in our institution during the period October 2018 until December 2020 (**Table S1**). Strains were isolated in blood agar and EMB agar media. The first identification was achieved by MALDI-TOF. Antibiotic susceptibility profiles were determined with the BD Phoenix system according to the guidelines and interpretation criteria of the Clinical and Laboratory Standards Institute (CLSI, 2022). Susceptibility to colistin was done by the pre-diffusion method according to the National Antimicrobial Reference Laboratory, Malbrán Institute, INEI-ANLIS, Argentina (http://antimicrobianos.com.ar/ATB/wp-content/uploads/2017/09/Protocolo-Predifusion-Tabletas-COL-Rosco-version2-Agosto2017.pdf).

Although fosfomycin breakpoint is only for *E. coli*, we extrapolated it to *K. pneumoniae* and the ECC. The KPC-producing *E. hormaechei* HAC11Eho (ST78), *E. hormaechei* HA58Eho (ST66), *K. pneumoniae* HA3pKpn (ST258), and *K. pneumoniae* HA15pKpn (ST11) strains from this survey were chosen for fitness assays.

### DNA sequencing and bioinformatic analysis

Genomic DNA extraction was performed using a QIAamp^®^ DNA Mini QIAcube Kit, and libraries were prepared with COVIDSeq Test (Illumina, San Diego, CA, USA) starting from the library preparation step. The libraries were sequenced at the Malbrán Institute in Argentina on Illumina MiSeq-I (Illumina, San Diego, CA, USA) with a MiSeq Reagent Kits v2 cartridge with 500 cycles in the case of HA2pEho and 300 in the case of HA7pKpn.

Quality inspection of the reads was performed using FASTQC v0.11.9 (Wingett and Andrews, 2018), while Trimmomatic v0.39 (Bolger et al., 2014) was used for adapter clipping and trimming low-quality reads. SPAdes v3.15.3 (Prjibelski et al., 2020) was used for genome assembly, and QUAST v5.0.2 was used for assessing the quality of the assembly (Quast et al., 2012). Prokka v1.14.5 was used for genome annotation (Seemann, 2014). Some of these tools were run at the European Galaxy server (Afgan et al., 2016). The number of reads for *E. hormaechei* HA2pEho was 1,355,790 with a length of 250 bp, and that for *K. pneumoniae* HA7pKpn was 1,542,461 with an average of 150 bp. The assembly size was 4,907,320 bp in 88 contigs for *E. hormaechei* HA2pEho and 5,581,104 in 161 contigs for *K. pneumoniae* HA7pKpn. The N50 was 327,017 for *E. hormaechei* HA2pEho and 181,947 for *K. pneumoniae* HA7pKpn.

Based on the information from WGS, further identification of the strains was carried out using several *in silico* molecular methods: rMLST (Jolley et al., 2018), Kraken 2 (which also analyzes the presence of contaminations; Wood et al., 2019), average nucleotide identity (ANI) (Jain et al., 2018), and *in silico* DNA–DNA hybridization (Meier-Kolthoff et al., 2022) (**Tables S8 and S9**). Whole-genome sequences of type strains were downloaded from the NCBI database based on the studies from Cho et al. (2021) and Wu et al. (2020).

Antibiotic resistance genes were searched using Resfinder (Bortolaia et al., 2020) and CARD (Alcock et al., 2019), and plasmids were found with PlasmidFinder. The identity of β-lactamases was refined using the β-lactamase database (Naas et al., 2017). Plasmids were further analyzed using BLAST (Altschu et al., 1990). The whole sequence of pDCCK1-KPC was on a single contig for both strains. The contiguousness of the extremes of the contig was verified by PCR using specially designed primers. In the case of HA2pEho, primers used were pDCCK1-F: CTGTACATGAAGGCGAAATGTCC and pDCCK1-R: CCTCATTCGTGCGCTCTAGG, and for HA7pKpn, the following primers were used: pDCCK-1B-F: GCGTGTAATGCAGATGGCAG and pDCCK-1B-R: ATGTATCTGCGTCCTGAGCG.

**Figures 1** and **2** were made using the ggplot2 (Alboukadel, 2018) package in R (R Core Team, 2020), and **Figure 3** was made using BRIG (Alikhan et al., 2011).

### Conjugation assays

Briefly, mid-log cultures of donor (HA2pEho and HA7pKpn) and recipient (*E. coli* J53) strains were mixed in LB broth (Laboratorios Britania S.A., Argentina). The mating culture was then incubated overnight at 37°C using the drop plate method. Four replicas were used in each conjugation: only LB, LB with the addition of meropenem (8 µg/ml), sodium azide (80 µg/ml), or a combination of both. To verify that colonies growing on both antibiotics were transconjugant *E. coli* J53, they were grown on EMB agar (Laboratorios Britania S.A., Argentina). *bla*_KPC-2_ PCR was carried out with the primers KPC-F: CCGTCAGTTCTGCTGTC and KPC-R: CGTTGTCATCCTCGTTAG (Ramírez et al., 2013) using GoTaq^®^ enzyme (Promega, Madison, WI).

### *In vitro* competition and fitness measurements

Growth rate and generation times were calculated by measuring DO at 600 nm. This was done in triplicate. All experiments involved in competition assays were carried out without selective pressure. To test plasmid stability in the absence of selective pressure, we carried out plasmid stability assays. This consisted of subculturing the isolates without antibiotics for 120 h. A single colony was picked from an agar plate containing 8 µg/ml of meropenem with the clone and left to grow overnight (ON) in 5 ml of LB media without the addition of antibiotics. After 24 h, 50 µl of ON culture was inoculated in 5 ml of LB media. After repeating this procedure four times, 100 µl of a 1E-07 dilution was plated on an LB agar plate, and 30 colonies were randomly picked. The presence of *bla*_KPC-2_ was determined by PCR.

For competitions, isolates were diluted to 1.6E+08 (OD 600 0.2) colony-forming units (CFU)/ml, and equal volumes were combined; thus, the initial ratio of the isolate pairs was close to 1:1; then, 10 μl of the mixture was added to 20 ml of LB broth and grown at 37°C with agitation at 180 rpm. At 24-h intervals, 10 μl of bacterial subcultures was transferred to 20 ml of fresh LB broth. This was done in triplicate. At 72 and 96 h, 100 μl was inoculated on EMB agar plates and left to grow ON at 37°C. The CFU of HA7pKpn and HA2pEho were counted, and after 96 h, the adaptive difference was calculated with the equation


$$\text{S}=\text{ln}\left[{\left(\frac{\frac{{\text{ECC}}_{\text{t}}}{{\text{Kpn}}_{\text{t}}}}{\frac{{\text{ECCt}}_{\text{t}-1}}{{\text{Kpn}}_{\text{t}-1}}}\right)}^{\frac{1}{\text{y}}}\right]$$

and fitness F = 1 + S, where S is the selection coefficient and shows the difference in fitness between two competing strains at time t, ECC_t_ = number of *E. hormaechei* colonies, Kpn_t_ = number of *K. pneumoniae* colonies, and ECC_t−1_ and Kpn_t−1_ are the number of ECC and *K. pneumoniae* at the previous time, respectively. The quotient of the ratios of the cell numbers was standardized with 1/y, where “y” is the number of bacterial generations during the assay (Sander et al., 2002; Guo et al., 2012). Here, the exponent was 1/9 because cell numbers were determined every nine generations. The terms Kpn_t_/Kpn_t−1_ and ECC_t_/ECC_t−1_ give the growth rates for *K. pneumoniae* and ECC strains, respectively. Hence, S is the natural logarithm of the quotient of the growth rates of the competing strains. S is positive if the ECC bacterial fitness is increased compared with that of the *K. pneumoniae* competitor strain. Sander et al. (2002) applied this method to make comparisons between resistant and susceptible strains of a single species. In this case, we did not count resistant bacteria but counted the CFU of the different strains in the absence of antibiotics, as colonies could be differentiated thanks to their different ability to ferment lactose. In the case where colonies could not be differentiated, we randomly picked 15 colonies from each plate and did PCR with primers specific for *K. pneumoniae* (Kaushik and Balasubramanian, 2012). We then calculated the frequency of each species and multiplied it by the CFU number to obtain the CFU number of each species. Statistical analysis was done using ANOVA with an alpha value of 0.05.

## Data availability statement

The data presented in the study is deposited in GenBank, accession number PRJNA841267.

## Author contributions

DC and MPQ contributed to the conception and design of the study. CAK, NGA, BF, LF-C, ASG, ND, and GC performed the experimental assays. CAK, VEA, BPM, ACdVL, MP, MGM, and JC carried out bioinformatics analysis. DC structured the work, wrote and coordinated the drafts of the manuscript, and did the final edition. CAK, MPQ, NGA, and DC wrote the Results section. All the authors contributed to the analysis of the data and manuscript revision, read, and approved the submitted version.

## Funding

MPQ and DC are members of the career investigator of CONICET, Argentina. CAK, AcdVL, and VEA have a Posdoctoral Fellowship from CONICET. MP is recipient of an Universidad de Buenos Aires´s doctoral fellowship and ASG has a Doctoral Fellowship from CONICET. NGA is an infectious diseases specialist. BF and LF-C are clinical microbiology specialists. Aires doctoral fellowship. NA is an infectious diseases doctor. BF and LF-C are clinical microbiology specialists. This study was supported by grants ISID/Pfizer 2019 (#56570859) and PUE 2522 from CONICET given to DC and IMPaM, respectively.

## Acknowledgments

We would like to thank Andrea Aguilar and Nicolás Mendiondo for their work on bioinformatic analysis.

## Conflict of interest

MPQ and DC are members of the career investigator of CONICET, Argentina. CAK and VEA have a Posdoctoral Fellowship from CONICET, and ASG have a Doctoral Fellowship from CONICET. NGA is an infectious diseases specialist. This study was supported by grants ISID/Pfizer 2019 (#56570859) and PUE 2522 from CONICET given to DC, and IMPaM, respectively. The funders were not involved in the study design, collection, analysis, interpretation of data, the writing of this article or the decision to submit it for publication. All authors declare no other competing interests.

## Publisher’s note

All claims expressed in this article are solely those of the authors and do not necessarily represent those of their affiliated organizations, or those of the publisher, the editors and the reviewers. Any product that may be evaluated in this article, or claim that may be made by its manufacturer, is not guaranteed or endorsed by the publisher.

## References

[B1] Afgan E. Baker D. van den Beek M. Blankenberg D. Bouvier D. Čech M.. (2016). The galaxy platform for accessible, reproducible and collaborative biomedical analyses: 2016 update. Nucleic Acids Res. 44, W3–W10. doi: 10.1093/nar/gkw343 27137889 PMC4987906

[B2] Alboukadel K. (2018) Ggpubr: “ggplot2” based publication ready plots. r package version 0.2. Available at: https://CRAN.R-project.org/package=ggpubr.

[B3] Alcock B. P. Raphenya A. R. Lau T. T. Y. Tsang K. K. Bouchard M. Edalatmand A.. (2019). CARD 2020: antibiotic resistome surveillance with the comprehensive antibiotic resistance database. Nucleic Acids Res. 48, gkz935. doi: 10.1093/nar/gkz935 PMC7145624 31665441

[B4] Alikhan N.-F. Petty N. K. Ben Zakour N. L. Beatson S. A. (2011). BLAST ring image generator (BRIG): simple prokaryote genome comparisons. BMC Genomics 12, 402. doi: 10.1186/1471-2164-12-402 21824423 PMC3163573

[B5] Altschu P. S. F. Gish W. Miller W. Myers E. W. Lipman D. J. (1990). Basic local alignment search tool. 8. J. Mol. Biol. 215 (3), 403–410. doi: 10.1016/S0022-2836(05)80360-2 2231712

[B6] Ávarez V. E. Quiroga M. P. Galán A. V. Vilacoba E. Quiroga C. Ramírez M. S.. (2020). Crucial Role of the Accessory Genome in the Evolutionary Trajectory of Acinetobacter baumannii Global Clone 1. Front. Microbiol. 11, 342. doi: 10.3389/fmicb.2020.00342 32256462 PMC7093585

[B7] Anchordoqui M. S. De Belder D. Lucero C. Rapoport M. Faccone D. Rodriguez A.. (2015). *In vivo* horizontal dissemination of the *bla* _KPC-2_ gene carried on diverse genetic platforms among clinical isolates of *Enterobacteriaceae* . J. Global Antimicrob. Resist. 3, 210–213. doi: 10.1016/j.jgar.2015.05.001 27873711

[B8] Annavajhala M. K. Gomez-Simmonds A. Uhlemann A.-C. (2019). Multidrug-resistant *Enterobacter cloacae* complex emerging as a global, diversifying threat. Front. Microbiol. 10. doi: 10.3389/fmicb.2019.00044 PMC6365427 30766518

[B9] BARNARDS Group Sands K. Carvalho M. J. Portal E. Thomson K. Dyer C.. (2021). Characterization of antimicrobial-resistant gram-negative bacteria that cause neonatal sepsis in seven low- and middle-income countries. Nat. Microbiol. 6, 512–523. doi: 10.1038/s41564-021-00870-7 33782558 PMC8007471

[B10] Bolger A. M. Lohse M. Usadel B. (2014). Trimmomatic: A flexible trimmer for illumina sequence data. Bioinformatics 30, 2114–2120. doi: 10.1093/bioinformatics/btu170 24695404 PMC4103590

[B11] Bonomo R. A. Burd E. M. Conly J. Limbago B. M. Poirel L. Segre J. A.. (2018). Carbapenemase-producing organisms: A global scourge. Clin. Infect. Dis. 66, 1290–1297. doi: 10.1093/cid/cix893 29165604 PMC5884739

[B12] Bortolaia V. Kaas R. S. Ruppe E. Roberts M. C. Schwarz S. Cattoir V.. (2020). ResFinder 4.0 for predictions of phenotypes from genotypes. J. Antimicrob. Chemother. 75, 3491–3500. doi: 10.1093/jac/dkaa345 32780112 PMC7662176

[B13] Botts R. T. Apffel B. A. Walters C. J. Davidson K. E. Echols R. S. Geiger M. R.. (2017). Characterization of four multidrug resistance plasmids captured from the sediments of an urban coastal wetland. Front. Microbiol. 8, 1922. doi: 10.3389/fmicb.2017.01922 29067005 PMC5641379

[B14] Brandt C. Viehweger A. Singh A. Pletz M. W. Wibberg D. Kalinowski J.. (2019). Assessing genetic diversity and similarity of 435 KPC-carrying plasmids. Sci. Rep. 9, 11223. doi: 10.1038/s41598-019-47758-5 31375735 PMC6677891

[B15] Campos L. C. Lobianco L. F. Seki L. M. Santos R. M. R. Asensi M. D. (2007). Outbreak of *Enterobacter hormaechei* septicaemia in newborns caused by contaminated parenteral nutrition in Brazil. J. Hosp. Infect. 66, 95–97. doi: 10.1016/j.jhin.2007.02.013 17428576

[B16] Center for Disease Dynamics, Economics & Policy ResistanceMap. Available at: https://resistancemap.cddep.org/About.php.

[B17] Chabbert Y. A. Scavizzi M. R. Witchitz J. L. Gerbaud G. R. Bouanchaud D. H. (1972). Incompatibility groups and the classification of *fi* ^–^ resistance factors. J. Bacteriol. 112, 666–675. doi: 10.1128/jb.112.2.666-675.1972 4628744 PMC251473

[B18] Cho G.-S. Stein M. Fiedler G. Igbinosa E. O. Koll L. P. Brinks E.. (2021). Polyphasic study of antibiotic-resistant enterobacteria isolated from fresh produce in Germany and description of enterobacter vonholyi sp. nov. isolated from marjoram and enterobacter dykesii sp. nov. isolated from mung bean sprout. System. Appl. Microbiol. 44, 126174. doi: 10.1016/j.syapm.2020.126174 33370657

[B19] CLSI (2022). Performance Standards for Antimicrobial Susceptibility Testing, supplement M100. 32nd ed. (USA: Clinical and Laboratory Standards Institute).

[B20] Davin-Regli A. Lavigne J.-P. Pagès J.-M. (2019). *Enterobacter* spp.: Update on taxonomy, clinical aspects, and emerging antimicrobial resistance. Clin. Microbiol. Rev. 32, e00002–19. doi: 10.1128/CMR.00002-19 31315895 PMC6750132

[B21] De Belder D. Lucero C. Rapoport M. Rosato A. Faccone D. Petroni A.. (2018). Genetic diversity of KPC-producing *Escherichia coli, klebsiella oxytoca, serratia marcescens*, and *Citrobacter freundii* isolates from Argentina. Microb. Drug Resist. 24, 958–965. doi: 10.1089/mdr.2017.0213 29236574

[B22] Dong D. Mi Z. Li D. Gao M. Jia N. Li M.. (2020). Novel IncR/IncP6 hybrid plasmid pCRE3-KPC recovered from a clinical KPC-2-Producing citrobacter braakii isolate. mSphere 5 ,e00891–19. doi: 10.1128/mSphere.00891-19 32213624 PMC7096625

[B23] Emeraud C. Petit C. Gauthier L. Bonnin R. A. Naas T. Dortet L. (2022). Emergence of VIM-producing enterobacter cloacae complex in France between 2015 and 2018. J. Antimicrob. Chemother. 77, 944–951. doi: 10.1093/jac/dkab471 35045171

[B24] Falco A. Guerrero D. García I. Correa A. Rivera S. Olaya M. B.. (2021). Molecular characterization of KPC-2-Producing *Enterobacter cloacae* complex isolates from cali, Colombia. Antibiotics 10, 694. doi: 10.3390/antibiotics10060694 34200675 PMC8229714

[B25] Fernández J. Montero I. Martínez Ó. Fleites A. Poirel L. Nordmann P.. (2015). Dissemination of multiresistant *Enterobacter cloacae* isolates producing OXA-48 and CTX-M-15 in a Spanish hospital. Int. J. Antimicrob. Agents 46, 469–474. doi: 10.1016/j.ijantimicag.2015.07.003 26307466

[B26] Frost I. Van Boeckel T. P. Pires J. Craig J. Laxminarayan R. (2019). Global geographic trends in antimicrobial resistance: the role of international travel. J. Travel Med. 26, taz036. doi: 10.1093/jtm/taz036 31115466

[B27] Ghiglione B. Haim M. S. Penzotti P. Brunetti F. D´Amico González G. Di Conza J.. (2021). Characterization of emerging pathogens carrying *bla* _KPC-2_ gene in IncP-6 plasmids isolated from urban sewage in Argentina. Front. Cell. Infect. Microbiol. 11. doi: 10.3389/fcimb.2021.722536 PMC8421773 34504809

[B28] Girlich D. Ouzani S. Emeraud C. Gauthier L. Bonnin R. A. Le Sache N.. (2021). Uncovering the novel *Enterobacter cloacae* complex species responsible for septic shock deaths in newborns: A cohort study. Lancet Microbe 2, e536–e544. doi: 10.1016/S2666-5247(21)00098-7 35544179

[B29] Gomez S. A. Pasteran F. G. Faccone D. Tijet N. Rapoport M. Lucero C.. (2011). Clonal dissemination of *Klebsiella pneumoniae* ST258 harbouring KPC-2 in Argentina. Clin. Microbiol. Infect. 17, 1520–1524. doi: 10.1111/j.1469-0691.2011.03600.x 21851480

[B30] Gomez-Simmonds A. Annavajhala M. K. Wang Z. Macesic N. Hu Y. Giddins M. J.. (2018). Genomic and geographic context for the evolution of high-risk carbapenem-resistant *Enterobacter cloacae* complex clones ST171 and ST78. mBio 9 ,e00542–18. doi: 10.1128/mBio.00542-18 29844109 PMC5974468

[B31] Guo B. Abdelraouf K. Ledesma K. R. Nikolaou M. Tam V. H. (2012). Predicting bacterial fitness cost associated with drug resistance. J. Antimicrob. Chemother. 67, 928–932. doi: 10.1093/jac/dkr560 22232512

[B32] Hafza N. Challita C. Dandachi I. Bousaab M. Dahdouh E. Daoud Z. (2018). Competition assays between ESBL-producing *E. coli* and *K. pneumoniae* isolates collected from Lebanese elderly: An additional cost on fitness. J. Infect. Public Health 11, 393–397. doi: 10.1016/j.jiph.2017.09.010 28988774

[B33] Hansen G. T. (2021). Continuous evolution: Perspective on the epidemiology of carbapenemase resistance among *Enterobacterales* and other gram-negative bacteria. Infect. Dis. Ther. 10, 75–92. doi: 10.1007/s40121-020-00395-2 33492641 PMC7954928

[B34] Heiden S. E. Hübner N.-O. Bohnert J. A. Heidecke C.-D. Kramer A. Balau V.. (2020). A *Klebsiella pneumoniae* ST307 outbreak clone from Germany demonstrates features of extensive drug resistance, hypermucoviscosity, and enhanced iron acquisition. Genome Med. 12, 113. doi: 10.1186/s13073-020-00814-6 33298160 PMC7724794

[B35] Hertz F. B. Marvig R. L. Frimodt-Møller N. Nielsen K. L. (2022). *In vitro* relative fitness, *in vivo* intestinal colonization and genomic differences of *Escherichia coli* of ST131 carrying *bla* _CTX–M–15_ . Front. Microbiol. 12. doi: 10.3389/fmicb.2021.798473 PMC8894762 35250906

[B36] Izdebski R. Baraniak A. Herda M. Fiett J. Bonten M. J. M. Carmeli Y.. (2015). MLST reveals potentially high-risk international clones of *Enterobacter cloacae* . J. Antimicrob. Chemother. 70, 48–56. doi: 10.1093/jac/dku359 25216820

[B37] Jain C. Rodriguez-R. L. M. Phillippy A. M. Konstantinidis K. T. Aluru S.. (2018). High throughput ANI analysis of 90K prokaryotic genomes reveals clear species boundaries. Nat. Commun. 9, 5114. doi: 10.1038/s41467-018-07641-9 30504855 PMC6269478

[B38] Jia X. Dai W. Ma W. Yan J. He J. Li S.. (2018). Carbapenem-resistant *E. cloacae* in southwest China: Molecular analysis of resistance and risk factors for infections caused by NDM-1-Producers. Front. Microbiol. 9. doi: 10.3389/fmicb.2018.00658 PMC5893741 29670607

[B39] Jolley K. A. Bray J. E. Maiden M. C. J. (2018). Open-access bacterial population genomics: BIGSdb software, the PubMLST.org website and their applications. Wellcome Open Res. 3, 124. doi: 10.12688/wellcomeopenres.14826.1 30345391 PMC6192448

[B40] Kaushik R. Balasubramanian R. (2012). Assessment of bacterial pathogens in fresh rainwater and airborne particulate matter using real-time PCR. Atmospheric Environ. 46, 131–139. doi: 10.1016/j.atmosenv.2011.10.013

[B41] Knecht C. A. Allende N. G. Álvarez V. E. Cormick B. P. M. Massó M. G. Campos J.. (2022). New sequence type of an enterobacter cloacae complex strain with the potential to become a high-risk clone. J. Global Antimicrob. Resist. 31, 162–164. doi: 10.1016/j.jgar.2022.08.015. S2213716522002065. 36049730

[B42] Luo N. Pereira S. Sahin O. Lin J. Huang S. Michel L.. (2005). Enhanced *in vivo* fitness of fluoroquinolone-resistant *Campylobacter jejuni* in the absence of antibiotic selection pressure. Proc. Natl. Acad. Sci. U.S.A. 102, 541–546. doi: 10.1073/pnas.0408966102 15634738 PMC545549

[B43] Magiorakos A.-P. Srinivasan A. Carey R. B. Carmeli Y. Falagas M. E. Giske C. G.. (2012). Multidrug-resistant, extensively drug-resistant and pandrug-resistant bacteria: An international expert proposal for interim standard definitions for acquired resistance. Clin. Microbiol. Infect. 18, 268–281. doi: 10.1111/j.1469-0691.2011.03570.x 21793988

[B44] Mathers A. J. Peirano G. Pitout J. D. D. (2015). The role of epidemic resistance plasmids and international high-risk clones in the spread of multidrug-resistant *Enterobacteriaceae* . Clin. Microbiol. Rev. 28, 565–591. doi: 10.1128/CMR.00116-14 25926236 PMC4405625

[B45] Meier-Kolthoff J. P. Carbasse J. S. Peinado-Olarte R. L. Göker M. (2022). TYGS and LPSN: A database tandem for fast and reliable genome-based classification and nomenclature of prokaryotes. Nucleic Acids Res. 50, D801–D807. doi: 10.1093/nar/gkab902 34634793 PMC8728197

[B46] Naas T. Oueslati S. Bonnin R. A. Dabos M. L. Zavala A. Dortet L.. (2017). Beta-lactamase database (BLDB) – structure and function. J. Enzyme Inhibition Medicinal Chem. 32, 917–919. doi: 10.1080/14756366.2017.1344235 PMC6445328 28719998

[B47] Neil K. Allard N. Grenier F. Burrus V. Rodrigue S. (2020). Highly efficient gene transfer in the mouse gut microbiota is enabled by the Incl2 conjugative plasmid TP114. Commun. Biol. 3, 523. doi: 10.1038/s42003-020-01253-0 32963323 PMC7508951

[B48] Otto M. (2013). Community-associated MRSA: What makes them special? Int. J. Med. Microbiol. 303, 324–330. doi: 10.1016/j.ijmm.2013.02.007 23517691 PMC3729626

[B49] Paauw A. Caspers M. P. M. Leverstein-van Hall M. A. Schuren F. H. J. Montijn R. C. Verhoef J.. (2009). Identification of resistance and virulence factors in an epidemic *Enterobacter hormaechei* outbreak strain. Microbiology 155, 1478–1488. doi: 10.1099/mic.0.024828-0 19372158

[B50] Peirano G. Matsumura Y. Adams M. D. Bradford P. Motyl M. Chen L.. (2018). Genomic epidemiology of global carbapenemase-producing enterobacter spp. 2008–2014. Emerg. Infect. Dis. 24, 1010–1019. doi: 10.3201/eid2406.171648 29774858 PMC6004858

[B51] Pitout J. D. D. Finn T. J. (2020). The evolutionary puzzle of *Escherichia coli* ST131. Infect. Genet. Evol. 81, 104265. doi: 10.1016/j.meegid.2020.104265 32112974

[B52] Prjibelski A. Antipov D. Meleshko D. Lapidus A. Korobeynikov A. (2020). Using SPAdes *De novo* assembler. Curr. Protoc. Bioinf. 70, e102. doi: 10.1002/cpbi.102 32559359

[B53] Quast C. Pruesse E. Yilmaz P. Gerken J. Schweer T. Yarza P.. (2012). The SILVA ribosomal RNA gene database project: improved data processing and web-based tools. Nucleic Acids Res. 41, D590–D596. doi: 10.1093/nar/gks1219 23193283 PMC3531112

[B54] Ramírez D. G. Nicola F. Zarate S. Relloso S. Smayevsky J. Arduino S. (2013). Emergence of *Pseudomonas aeruginosa* with KPC-type carbapenemase in a teaching hospital: An 8-year study. J. Med. Microbiol. 62, 1565–1570. doi: 10.1099/jmm.0.059923-0 23831767

[B55] R Core Team (2020). R: A language and environment for statistical computing (Vienna, Austria: R Foundation for Statistical Computing). Available at: https://www.R-project.org/.

[B56] Red WHONET Argentina Vigilancia de la resistencia a los antimicrobianos red WHONET Argentina 2010-2021. Available at: https://antimicrobianos.com.ar (Accessed May 10, 2022).

[B57] Sander P. Springer B. Prammananan T. Sturmfels A. Kappler M. Pletschette M.. (2002). Fitness cost of chromosomal drug resistance-conferring mutations. Antimicrob. Agents Chemother. 46, 1204–1211. doi: 10.1128/AAC.46.5.1204-1211.2002 11959546 PMC127173

[B58] Seemann T. (2014). Prokka: rapid prokaryotic genome annotation. Bioinformatics 30, 2068–2069. doi: 10.1093/bioinformatics/btu153 24642063

[B59] Shen P. Wei Z. Jiang Y. Du X. Ji S. Yu Y.. (2009). Novel genetic environment of the carbapenem-hydrolyzing β-lactamase KPC-2 among *Enterobacteriaceae* in China. Antimicrob. Agents Chemother. 53, 4333–4338. doi: 10.1128/AAC.00260-09 19620332 PMC2764158

[B60] Sidjabat H. E. Townell N. Nimmo G. R. George N. M. Robson J. Vohra R.. (2015). Dominance of IMP-4-Producing *Enterobacter cloacae* among carbapenemase-producing *Enterobacteriaceae* in Australia. Antimicrob. Agents Chemother. 59, 4059–4066. doi: 10.1128/AAC.04378-14 25918153 PMC4468659

[B61] Tavares C. P. Pereira P. S. Marques E. de A. Faria C. de Souza M.. (2015). Molecular epidemiology of KPC-2–producing *Enterobacteriaceae* (non–*Klebsiella pneumoniae*) isolated from Brazil. Diagn. Microbiol. Infect. Dis. 82, 326–330. doi: 10.1016/j.diagmicrobio.2015.04.002 25935630

[B62] Voth D. E. Broederdorf L. J. Graham J. G. (2012). Bacterial type IV secretion systems: versatile virulence machines. Future Microbiol. 7, 241–257. doi: 10.2217/fmb.11.150 22324993 PMC3563059

[B63] Wang Q. Zhang Y. Yao X. Xian H. Liu Y. Li H.. (2016). Risk factors and clinical outcomes for carbapenem-resistant *Enterobacteriaceae* nosocomial infections. Eur. J. Clin. Microbiol. Infect. Dis. 35, 1679–1689. doi: 10.1007/s10096-016-2710-0 27401905

[B64] Wingett S. Andrews S. (2018). FastQ screen: A tool for multi-genome mapping and quality control. F1000Res 7. doi: 10.12688/f1000research.15931.2 PMC6124377 30254741

[B65] Wiser M. J. Lenski R. E. (2015). A comparison of methods to measure fitness in *Escherichia coli* . PloS One 10, e0126210. doi: 10.1371/journal.pone.0126210 25961572 PMC4427439

[B66] Wood D. E. Lu J. Langmead B. (2019). Improved metagenomic analysis with kraken 2. Genome Biol. 13, 257–270. doi: 10.1186/s13059-019-1891-0 PMC6883579 31779668

[B67] World Health Organization (2017) WHO publishes list of bacteria for which new antibiotics are urgently needed. Available at: https://www.who.int/news/item/27-02-2017-who-publishes-list-of-bacteria-for-which-new-antibiotics-are-urgently-needed.

[B68] Wozniak A. Figueroa C. Moya-Flores F. Guggiana P. Castillo C. Rivas L.. (2021). A multispecies outbreak of carbapenem-resistant bacteria harboring the *bla* _KPC_ gene in a non-classical transposon element. BMC Microbiol. 21, 107. doi: 10.1186/s12866-021-02169-3 33836654 PMC8034096

[B69] Wu W. Feng Y. Zong Z. (2020). Precise species identification for *Enterobacter*: A genome sequence-based study with reporting of two novel species, *Enterobacter quasiroggenkampii* sp. nov. and *Enterobacter quasimori* sp. nov. mSystems 5, e00527–e00520. doi: 10.1128/mSystems.00527-20 32753511 PMC7406230

