# Supplementary material for: Novel insights related to the rise of KPC-producing Enterobacter cloacae complex strains within the nosocomial niche
Source: Front Cell Infect Microbiol. 2022 Oct 24;12:951049. doi: 10.3389/fcimb.2022.951049 (PMC9686827; doi:10.3389/fcimb.2022.951049)
Supplement: Supplementary file 1 [file DataSheet_1.pdf]

Supplementary figures for

"Novel insights related to the rise of KPC-producing Enterobacter cloacae Complex strains within the nosocomial niche"

Camila A. Knecht, Natalia García Allende, Verónica E. Álvarez, Bárbara Prack Mc Cormick, Mariana G. Massó, María Piekar, Josefina Campos, Barbara Fox, Gabriela Camicia, Anahí S. Gambino, Ana del Valle Carolina Leguina, Nicolás Donis, Liliana Fernández Canigia, María Paula Quiroga, Daniela Centrón

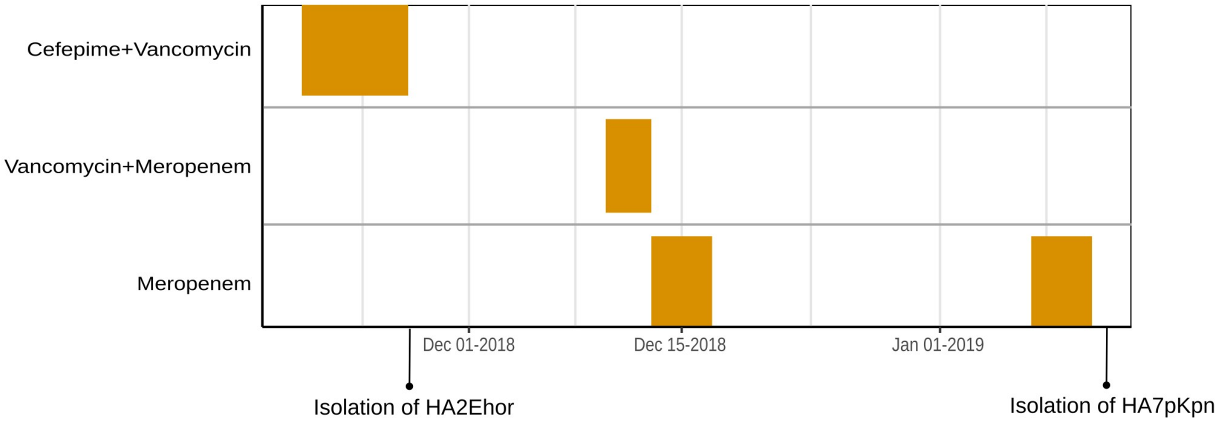

Figure S1| Timeline showing antibiotic treatments of inpatient M71 and isolation of colonising bacteria.

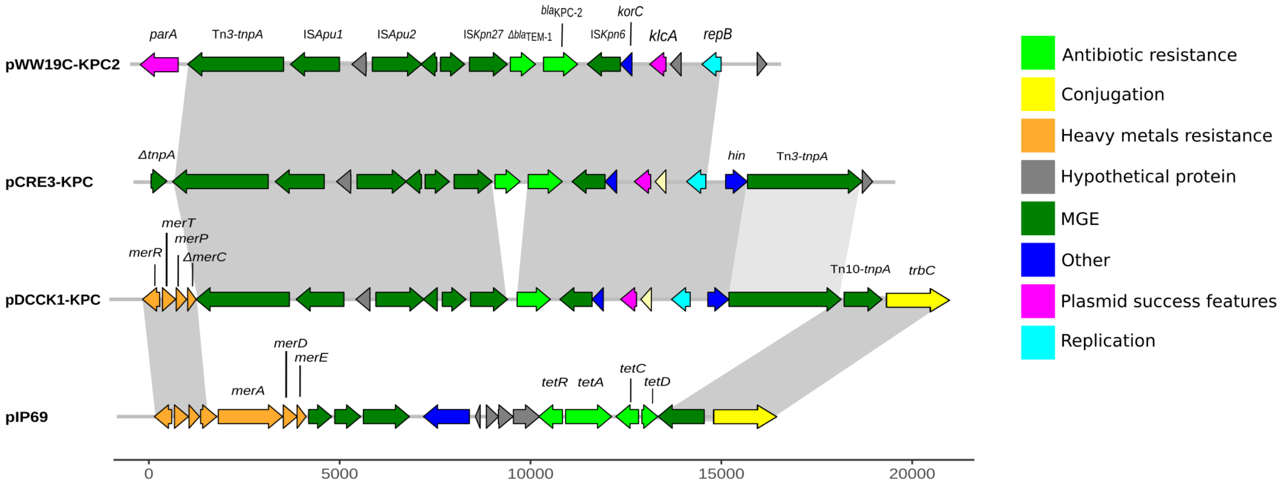

Figure S2| Genetic context of the *bla*$_{KPC-2}$ gene in the conjugative plasmid pDCCK1-KPC. The genetic platform in pDCCK1-KPC compared to similar platforms and with the plasmid pIP69 at the region where recombination might have occurred. Dark grey represents 100% of identity and light grey 75%. pWW19C-KPC2 (CP080110.1), pCRE3-KPC (MH919378.1) and pIP69 (MN626603.1).
